# Supplementary material for: Event-related functional MRI of awake behaving pigeons at 7T
Source: Nat Commun. 2020 Sep 18;11:4715. doi: 10.1038/s41467-020-18437-1 (PMC7501281; doi:10.1038/s41467-020-18437-1)
Supplement: Supplementary file 1 — Supplementary Information [file 41467_2020_18437_MOESM1_ESM.pdf]

Supplementary Information

Event-related functional MRI of awake behaving pigeons at 7T

Behroozi et al

Supplementary Note 1. Additional procedures to disentangle the neural activity of different components during a Go/NoGo task

We demonstrated that our experimental setup is well suited to reveal neural activation patterns during a visually guided Go/NoGo task. Future studies could differentiate further neurocognitive components by some modifications of the current protocol. As an example: Processing of the Go stimulus consists of the following steps: encoding of the stimulus; reward-based stimulus-response association, decision making, and motor output. In addition, associative learning studies demonstrated that reward expectation can severely influence the representation of sensory stimuli [1,2]. For example, if a Go stimulus reward has been coupled with a specific smell and/or sound, a pure visual presentation of the Go stimulus alone can elicit activation in the auditory, olfactory and somatosensory systems even if no auditory/olfactory stimuli are present and the reward ingestion has not begun yet.

On the other hand, the processing of NoGo stimuli mostly requires the relevant sensory system for stimulus encoding and inhibitory systems to prevent the animals' response.

The method of choice to differentiate between different components in an fMRI data set is the subtraction method. Using Go vs. NoGo contrast (as we did in the current study) is well suited to identify active brain regions during the Go stimulus phase, which, as we have shown, are located in the motor execution, decision making, reward expectation and possibly further sensory areas such as somatosensory (due to ingestion), auditory (because of extra sound) or olfactory (reward smell) systems, which were associated with the reward. Disentangling different components of these systems is much more challenging and requires changing the Go/NoGo paradigm in a way that either enables modeling of each component separately or balances events in both Go and NoGo trials. In the current study, we found that animals were mandibulating randomly during the ITI. By subtracting the activation pattern recorded during random mandibulations from the Go activation pattern, we were able to eliminate the motor component (beak movement) from our results. In addition, we designed our experiment in a way that the reward was only delivered after the Go stimulus offset to disassociate the ingestion effect.

To eliminate reward associated components like reward smell or sound during reward presentation as well as reward expectation, we would suggest a symmetrically reward Go/NoGo experiment [3]. This experiment is more or less identical to the paradigm used in our study with the exception that reward-associated sensory stimuli as well as factors like reward expectation are present in both Go and NoGo trials and can thus be subtracted from the data. The remaining activity pattern should thus solely represent the decision-making component.

Supplementary Methods

Since in FSL software the user has no access to change double-gamma HRF modeling parameters,

she/he should change these parameters based on pigeon HRF in the source distribution and then compile it.

1- Download the source distribution here: https://fsl.fmrib.ox.ac.uk/fsldownloads_registration

2- Change the double-gamma modeling parameters as follow:

-go to FSL/src/feat5 and read feat_model.cc

-replace the folowing lines:

1190 float sigma1=2.449, delay1=6, // first gamma

1191 sigma2=4, delay2=16, // second gamma

1192 ratio=6; // hrf = gammapdf1 - gammapdf2/ratio;

with these lines:

float sigma1=1.59, delay1=4.42, // first gamma

sigma2=4.58, delay2=15.5, // second gamma

ratio=4; // hrf = gammapdf1 - gammapdf2/ratio;

and save after applying changes.

3- You can compile the source distribution as described here:

https://fsl.fmrib.ox.ac.uk/fsl/fslwiki/FslInstallation/SourceCode

Supplementary Tables

Supplementary Table 1. Average plasma corticosterone values (ng per ml) ± SEM for four individual pigeons on different habituation days.

	**Day1 0 Minutes**	**Day1 10 Minutes**	**Day4 0 Minutes**	**Day4 10 Minutes**	**Day7 0 Minutes**	**Day7 10 Minutes**
**P_{Cort} 1**	247.8 ± 11.3	258.8 ± 12.8	213.9 ± 9.7	196.8 ± 7.7	250.5 ± 14.0	241.9 ± 13.8
**Change Δ**		11.1		-17.1		-8.5
**P_{Cort} 2**	110.6 ± 3.35	117.8 ± 6.0	120.1 ± 4.1	115.0 ± 5.1	123.3 ± 5.9	126.2 ± 4.7
**Change Δ**		7.3		-5.1		2.9
**P_{Cort} 3**	56.2 ± 3.7	85.3 ± 5.1	72.0 ± 1.8	76.0 ± 5.3	67.2 ± 7.0	68.9 ± 6.2
**Change Δ**		29.2		4.0		1.7
**P_{Cort} 4**	47.2 ± 3.0	68.8 ± 3.37	59.2 ± 4.6	67.3 ± 3.2	72.3 ± 6.2	76.4 ± 7.0
**Change Δ**		21.6		8.2		4.1
**Average Δ**		17.3 ± 4.99		-2.5 ± 5.6		0.1 ± 2.9

Supplementary Table 2. List of subjects involved in the different experiments.

Subject ID	Color Discrimination	Visual	Resting State	Stress
**P03**	✗	✓	✗	✗
**P04**	✗	✓	✗	✗
**P59**	✗	✓	✗	✗
**P60**	✗	✓	✗	✗
**P768**	✗	✓	✗	✗
**P05**	✓	✗	✓	✗
**P06**	✓	✗	✓	✗
**P07**	✓	✗	✓	✗
**P10**	✓	✗	✓	✗
**P53**	✓	✗	✓	✗
**P54**	✓	✗	✓	✗
**P55**	✓	✗	✓	✗
**P62**	✓	✗	✓	✗
**P401**	✗	✗	✗	✓
**P402**	✗	✗	✗	✓
**P403**	✗	✗	✗	✓
**P404**	✗	✗	✗	✓
**P501**	✗	✗	✗	✓
**P502**	✗	✗	✗	✓
**P503**	✗	✗	✗	✓

Supplementary Table 3. Alphabetized list of abbreviations

AA	Anterior arcopallium	MD	Mesopallium dorsale
Ac	N. accumbens	MFD	Mesopallium frontodorsale
AD	Arcopallium dorsale	MFV	Frontoventral mesopallium
AI	Arcopallium intermedium	MID	Dorsal intermediate mesopallium
AI	Arcopallium intermedium	MIVl	Mesopallium intermedioventrale, pars lateralis
AM	Arcopallium mediale	MIVm	Mesopallium intermedioventrale, pars medialis
AP	Posterior arcopallium	MOT	Medial olfactory tract
AV	Arcopallium ventrale	NCC	Central caudal nidopallium
VTA	Ventral tegmental area	NCLl	Lateral part of nidopallium caudolaterale
Bas	N. basalis prosencephali	NCLm	Medial part of nidopallium caudolaterale
BSTL	Bed nucleus of the stria terminalis, pars lateralis	NCM	Nidopallium caudomediale
BO	Bulbus olfactorius	NDB	Diagonal band of Broca
CDL	Area corticoidea dorsolateralis	NFL	Nidopallium frontolaterale
CM	Mesopallium caudale	NFT	nidopallium frontotrigeminale
CPP	Cortex prepiriformis	NIL	Nidopallium intermedium laterale
DLd	Dorsal part of dorsolateral region	NIMl	Nidopallium intermedium mediale pars lateralis
DLv	Ventral part of dorsolateral region	NMm	Nidopallium mediale pars medialis
DMv	Ventral part of the dorsomedial region	PoA	N. posterioris amygdalopallii pars basalis
DMd	Dorsal part of the dorsomedial region	SL	Septum laterale
E	Entopallium	SM	Septum mediale
GP	Globus pallidus	SpA	Area subpallialis amygdalae
HA	Hyperpallium apicale	StL	Striatum laterale
HbL	Lateral habenula	StM	Striatum mediale
HD	Hyperpallium dorsale	TnA	N. taeniae amygdalae
HI	Hyperpallium intercalatum	TPO	Area temporo-parieto-occipitalis
IHA	Interstitial nucleus of hyperpallium apicale	Tr	Triangular region of the ventromedial region
L1	Field L1	TuO	Tuberculum olfactorium
L2	Field L2	Vl	Ventrolateral part of the V-complex
L3	Field L3	Vm	Ventromedial part of the V-complex
MC	Mesopallium caudale	VP	Ventral pallidum

Supplementary Figures

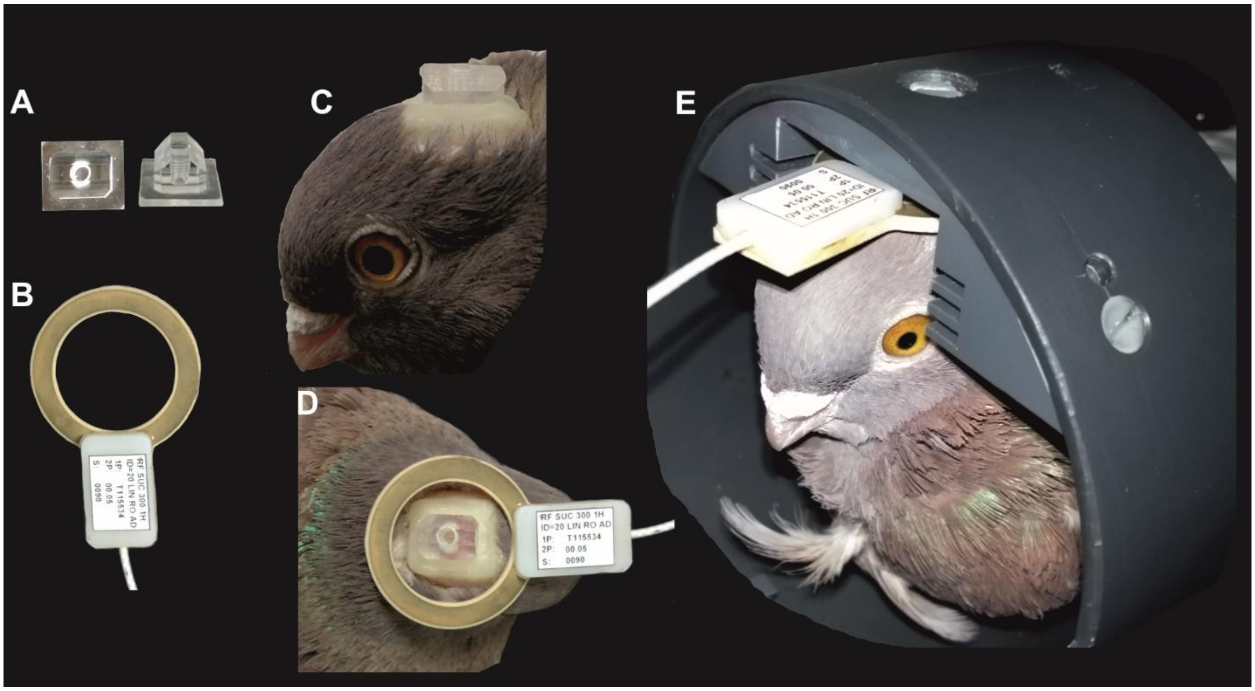

Supplementary Figure 1. A custom-made pigeon restrainer**.** (A) MRI-compatible pedestal for head fixation. (B) 20mm surface coil. (C) Lateral view of implanted pigeon with an MRI compatible pedestal. (D) Position of surface coil. (E) Fixated pigeon in custom made restrainer. First, the coil was positioned then the head was screwed to the body of restrainer via the implanted pedestal.

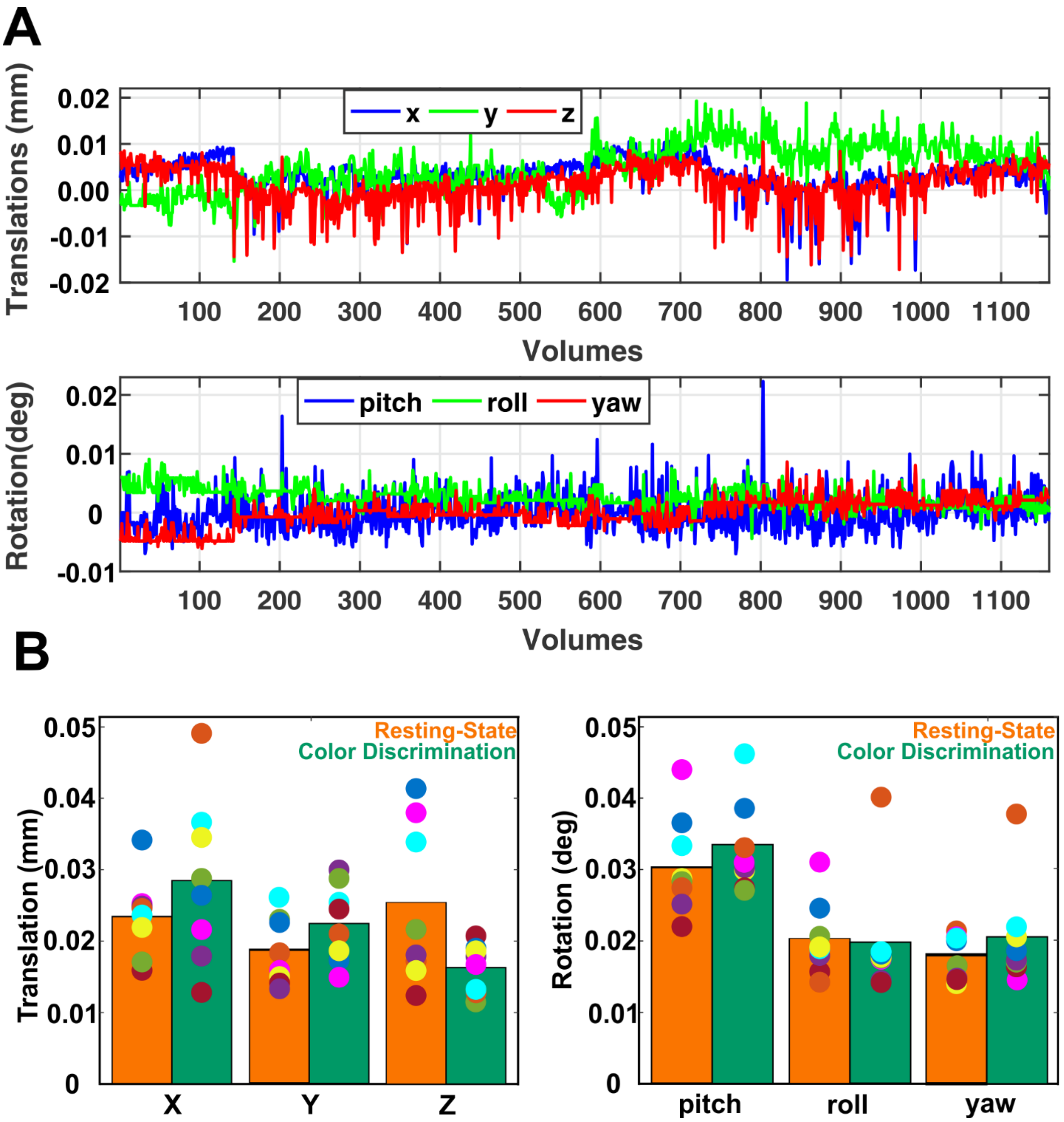

Supplementary Figure 2. Estimated motion parameters of an individual pigeon during active color discrimination. (A) The top row shows the translational (in mm), bottom row rotational movement parameters (in deg.) of a single individual. Parameters were estimated by a 3D rigid body model with six degrees of freedom for translation (x, y, and z-direction) and rotation (pitch, roll, and yaw). (B) Mean of maximum absolute rotation and translation parameters over all pigeons (n=8) during resting-state and color discrimination experiments. Circles represent single individuals. Source data are provided as a Source Data file.

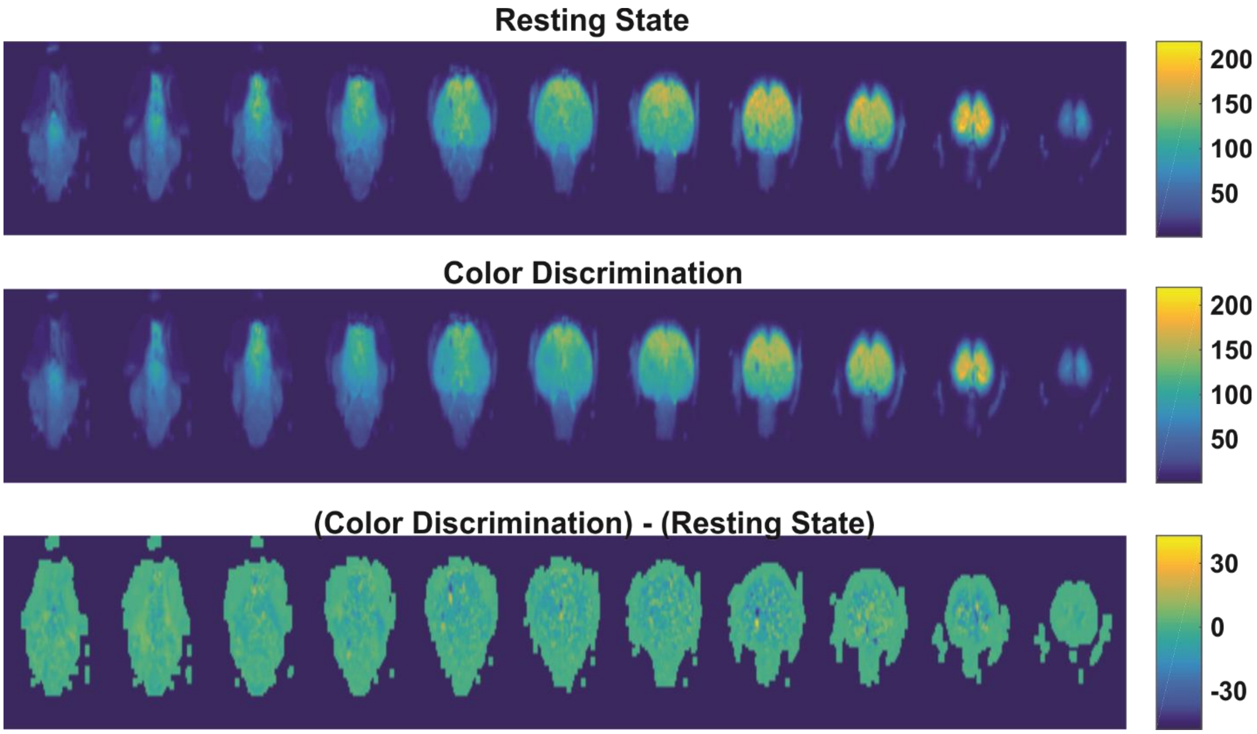

Supplementary Figure 3. Assessment of data quality. Temporal SNR (tSNR) maps for the 10 min resting-state scan (top row), the color discrimination task (middle row), and the difference between resting state and color discrimination task (bottom row). The tSNR values of the resting-state scan were used to calculate tSNR as the ratio of the time-series' mean and standard deviation, preceded by spatial smoothing and temporal filtering. The tSNR maps in the first two rows are highly similar indicating that mandibulation does not influence the quality of the fMRI images.

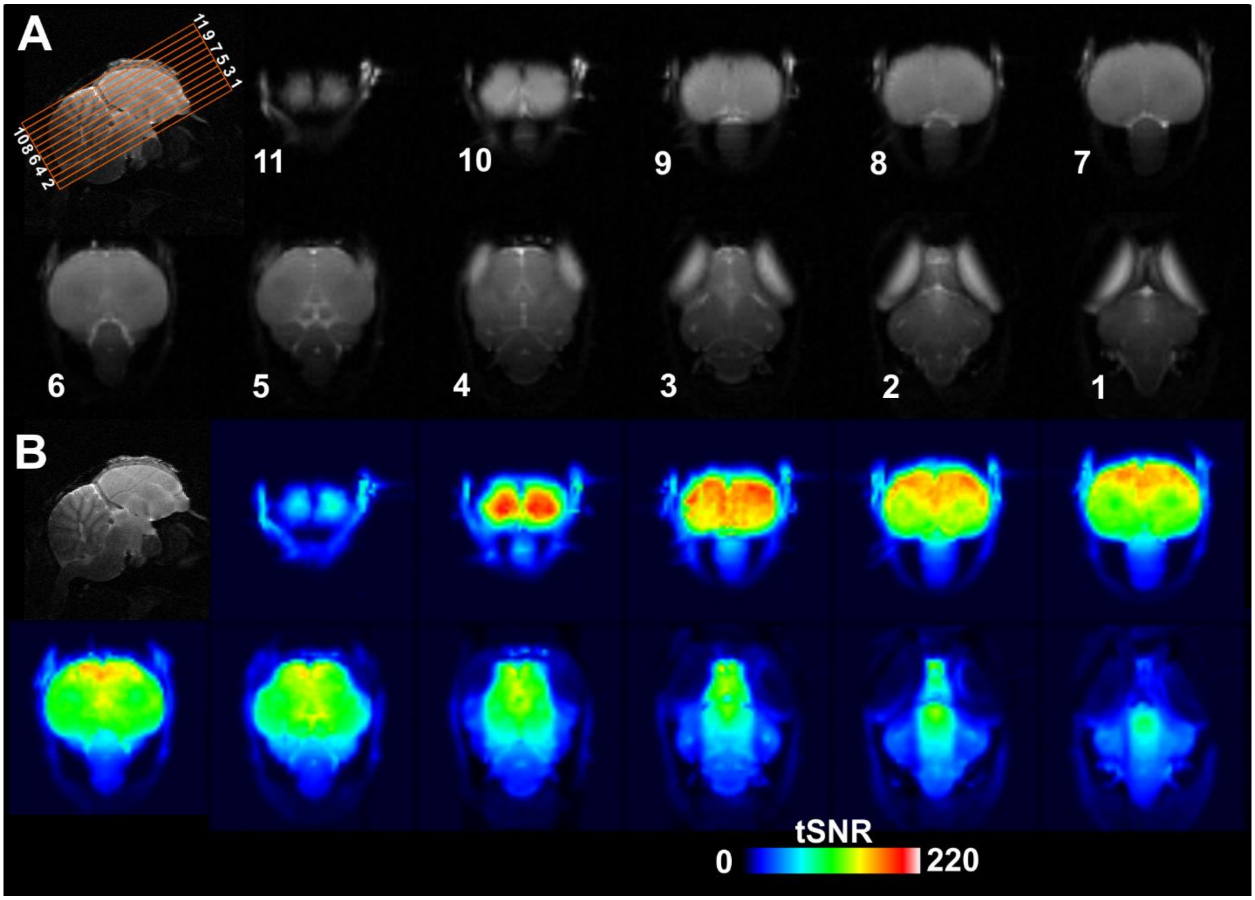

Supplementary Figure 4. Temporal signal to noise ratio**.** (A) Representative functional images acquired using multi-slice single-shot RARE sequence data from a single subject. (B) Temporal SNR maps, 10 min resting-state scan was used to calculate tSNR as the ratio of the time-series' mean and standard deviation, followed by spatial smoothing and temporal filtering. This figure illustrates that the tSNR (>100) is high in the regions of interest.

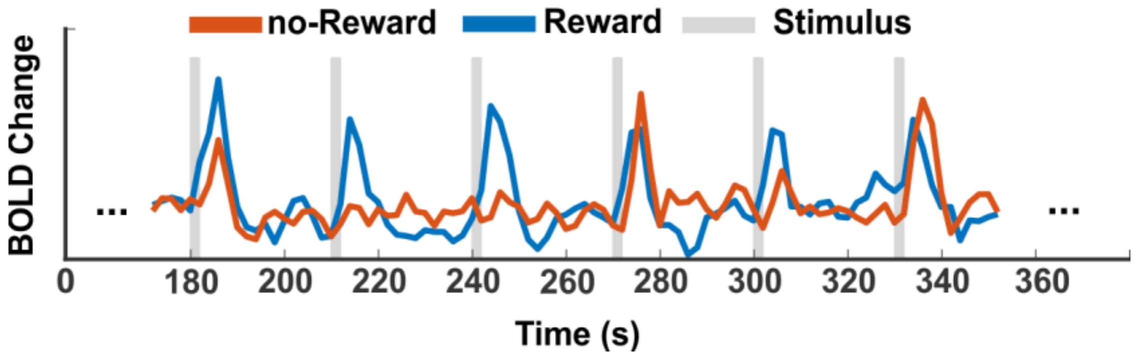

Supplementary Figure 5. Typical BOLD responses of the entopallium following visual stimulation. Since animals were rather relaxed during the visual experiment and tended to fall asleep in the dark scanner environment, they tended to miss trials. To measure reproducible BOLD signals over consecutive trials we thus offered a water reward to increase their attention to the task (blue line). This was confirmed additionally by visual inspection of the fMRI images.

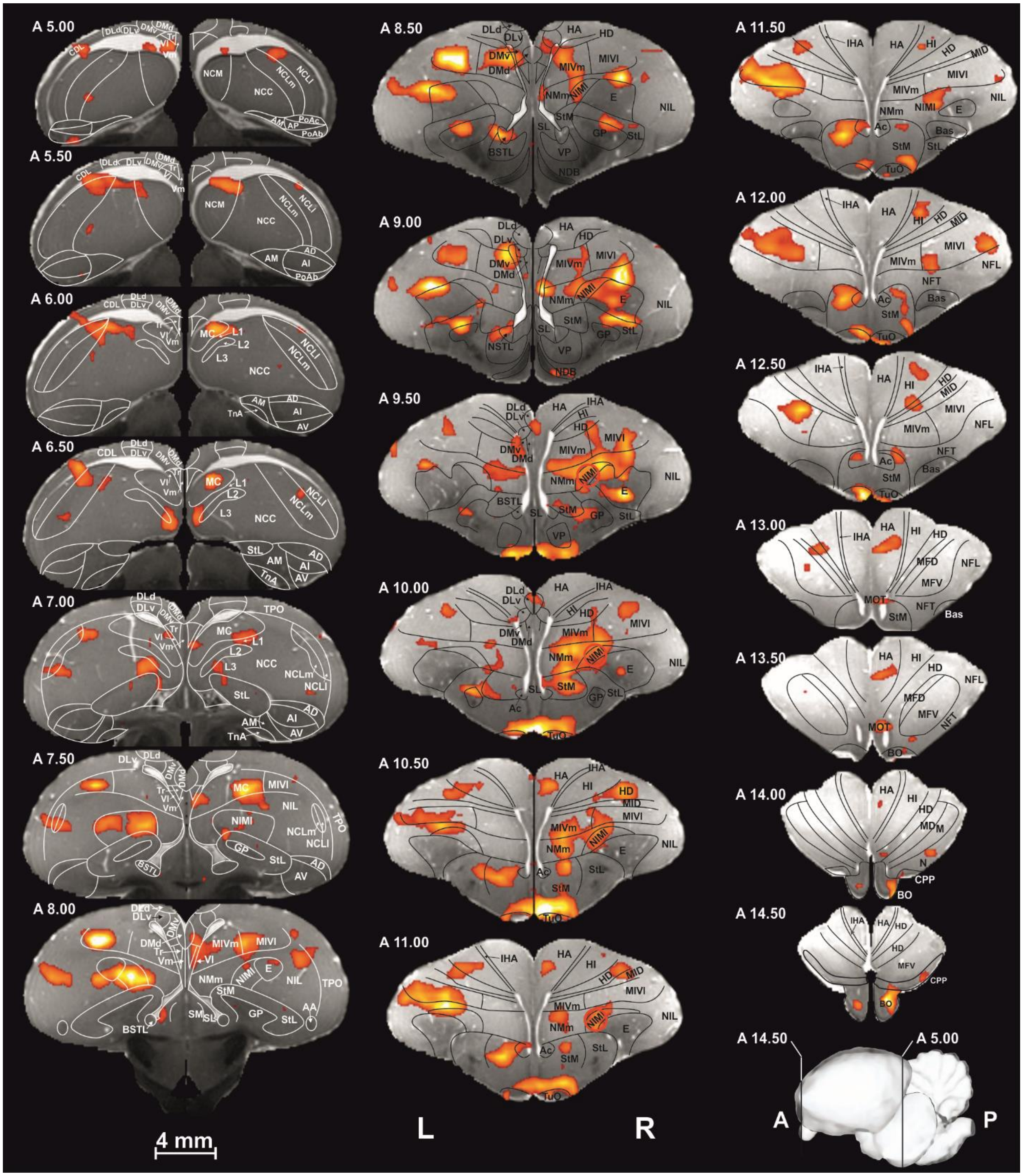

Supplementary Figure 6. BOLD response pattern during the color discrimination task. Statistical activation maps for the signal increase in the contrast of Go > NoGo + mandibulation (Z = 3.1 and $p < 0.05$ FWE corrected at the cluster level, group analysis). The activation maps were registered and illustrated on the pigeon atlas. The activation significance is demonstrated by the color scale. For abbreviation see caption of Figure 3.

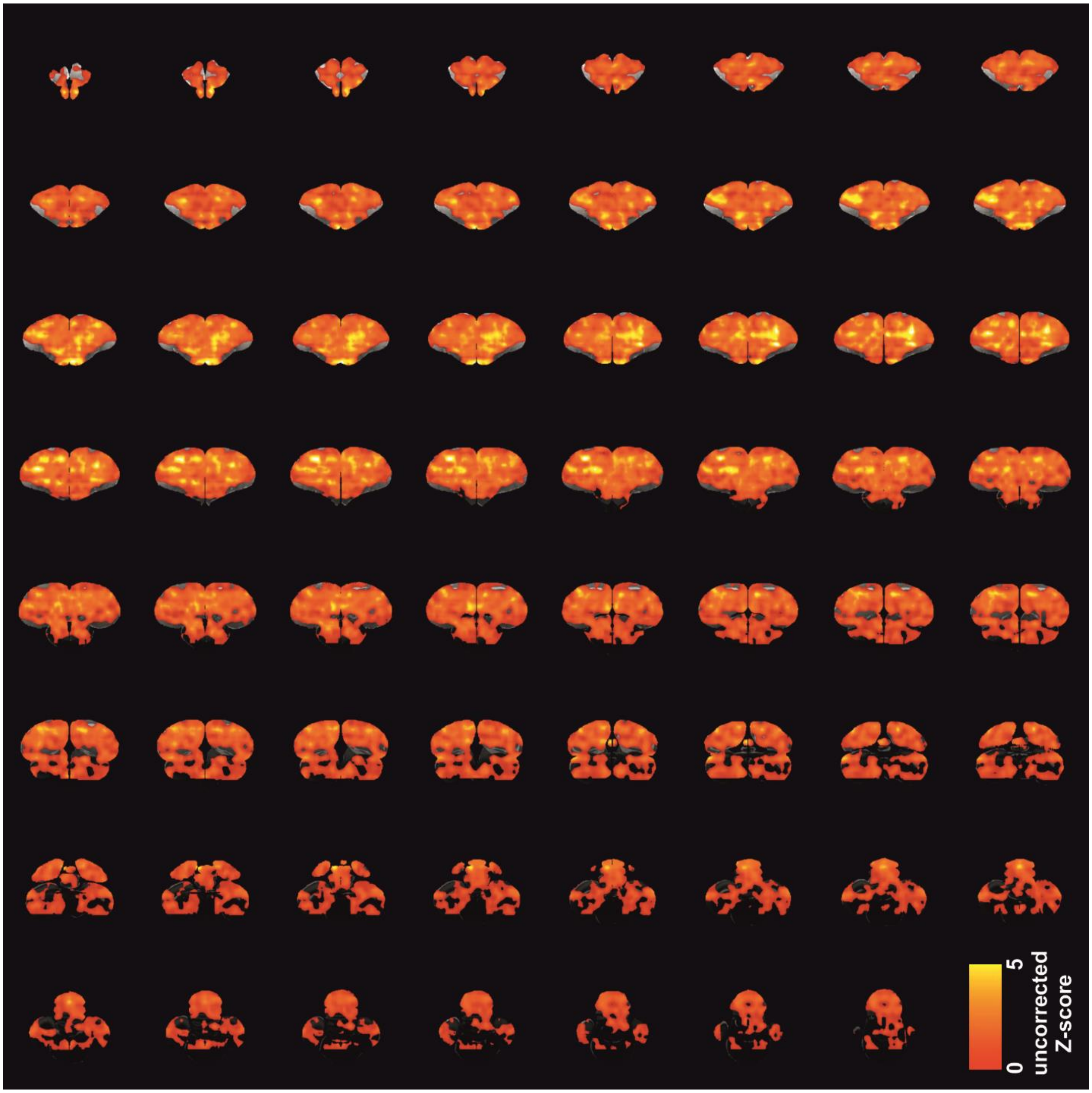

Supplementary Figure 7. Uncorrected and unthresholded statistical activation maps for the signal increase in the contrast of Go > NoGo + mandibulation.

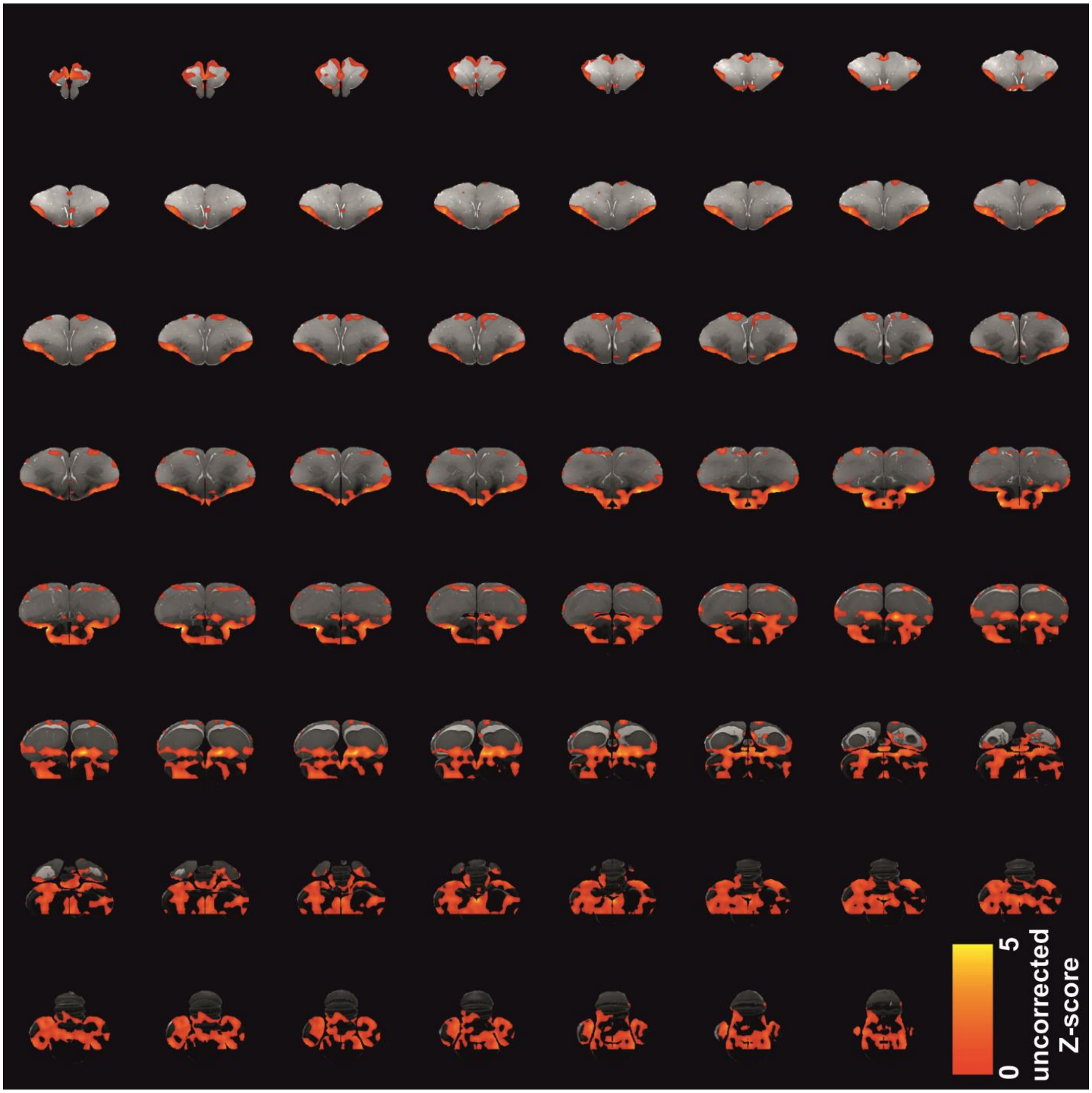

Supplementary Figure 8. Uncorrected and unthresholded statistical activation maps for the signal increase in the contrast of NoGo > Go

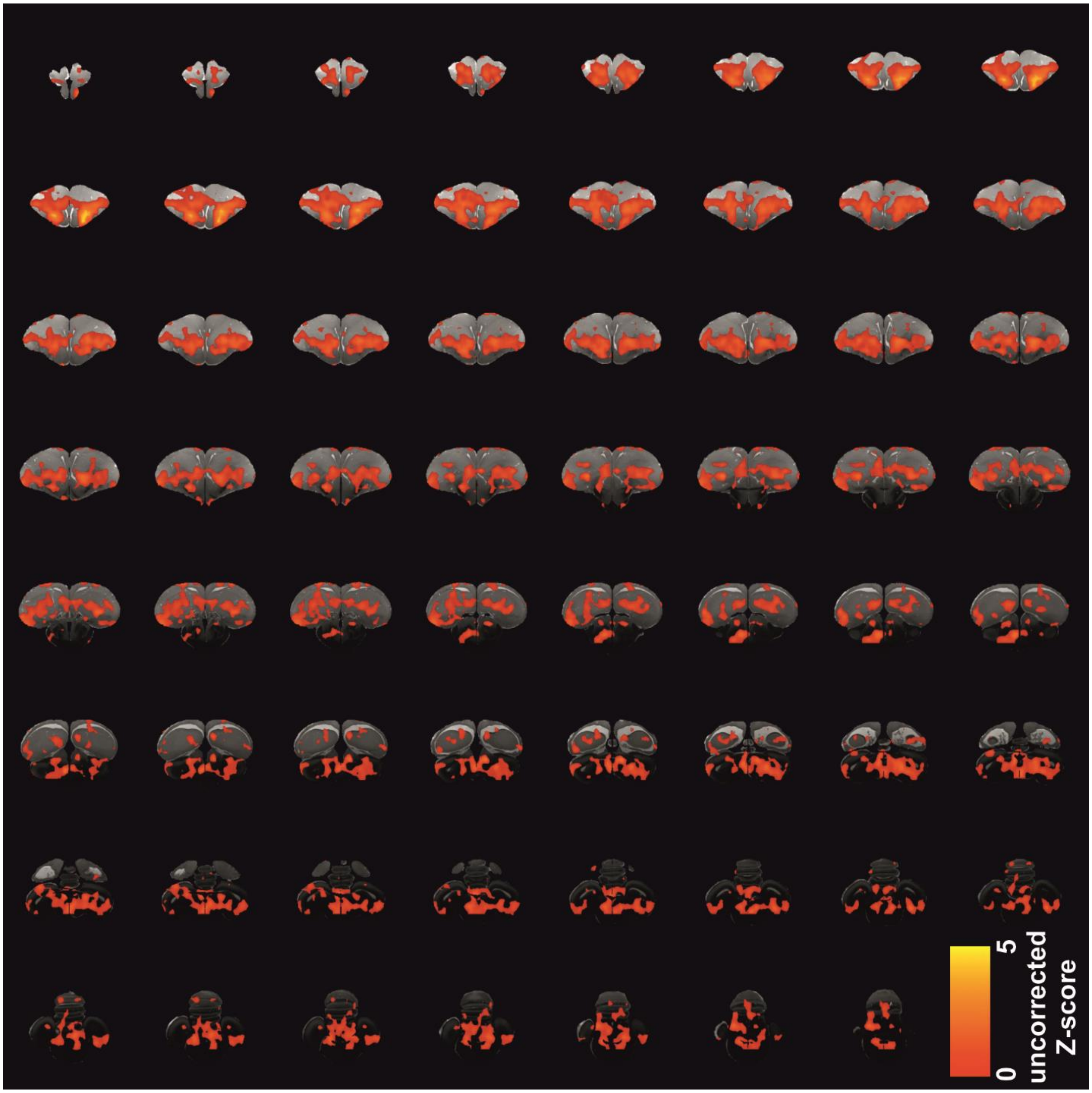

Supplementary Figure 9. Uncorrected and unthresholded statistical activation maps for the signal increase in the contrast of mandibulation > baseline.

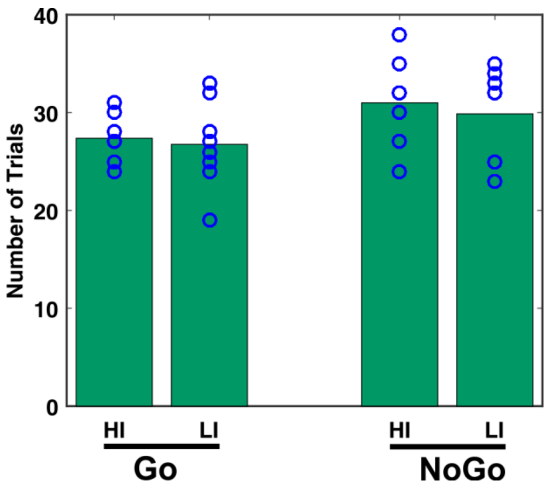

Supplementary Figure 10. Evaluating light intensity effect. The number of trials during which animals responded correctly over different light intensities during Go and NoGo trials (n = 8 pigeons). For Go trials, the number of trials with correct mandibulations is plotted, while for NoGo trials, trials with correct rejections are given. HI: high intensity; LI: low intensity.

Supplementary References

1. Zhou, Y. Di & Fuster, J. M. Visuo-tactile cross-modal associations in cortical somatosensory cells. *Proc. Natl. Acad. Sci. U. S. A.* **97**, 9777–9782 (2000).

2. Meyer, K. *et al.* Predicting visual stimuli on the basis of activity in auditory cortices. *Nat. Neurosci.* **13**, 667–668 (2010).

3. Syed, E. C. J. *et al.* Action initiation shapes mesolimbic dopamine encoding of future rewards. *Nat. Neurosci.* **19**, 34–36 (2015).
